# Fibrin Gel as an Injectable Biodegradable Scaffold and Cell Carrier for Tissue Engineering

**DOI:** 10.1155/2015/685690

**Published:** 2015-03-17

**Authors:** Yuting Li, Hao Meng, Yuan Liu, Bruce P. Lee

**Affiliations:** Department of Biomedical Engineering, Michigan Technological University, Houghton, MI 49931, USA

## Abstract

Due to the increasing needs for organ transplantation and a universal shortage of donated tissues, tissue engineering emerges as a useful approach to engineer functional tissues. Although different synthetic materials have been used to fabricate tissue engineering scaffolds, they have many limitations such as the biocompatibility concerns, the inability to support cell attachment, and undesirable degradation rate. Fibrin gel, a biopolymeric material, provides numerous advantages over synthetic materials in functioning as a tissue engineering scaffold and a cell carrier. Fibrin gel exhibits excellent biocompatibility, promotes cell attachment, and can degrade in a controllable manner. Additionally, fibrin gel mimics the natural blood-clotting process and self-assembles into a polymer network. The ability for fibrin to cure *in situ* has been exploited to develop injectable scaffolds for the repair of damaged cardiac and cartilage tissues. Additionally, fibrin gel has been utilized as a cell carrier to protect cells from the forces during the application and cell delivery processes while enhancing the cell viability and tissue regeneration. Here, we review the recent advancement in developing fibrin-based biomaterials for the development of injectable tissue engineering scaffold and cell carriers.

## 1. Introduction

According to the report by the U.S. Department of Health & Human Services, in 2013, there were over 121,000 patients waiting in the tissue donation list but there were only 14,000 donors and this gap continues to widen [1]. Due to the increasing needs for organ transplantation and a universal donor shortage, tissue engineering emerges as a useful approach to address this problem. Tissue engineering combines living cells and a suitable polymeric scaffold to regenerate functional tissues or organs. An ideal scaffold should be easy to handle, nontoxic or having no immunogenic effect, and showing good mechanical and chemical properties, as well as having controllable degradation to match the tissue development [2]. Although synthetic polymers such as polyglycolic acid, polylactic acid, and polyurethanes are widely used to fabricate tissue engineering scaffolds, these synthetic materials are limited by biocompatibility concerns, the inability to support cell attachment, toxic degradation products, and undesirable degradation rate [3, 4].

For biopolymer-based tissue engineering scaffolds, protein-based (i.e., fibrin, collagen) materials provide binding sites for cell adhesion, while the polysaccharide-based (i.e., alginate, chitosan, and agarose) scaffolds usually require further cell-attachment modification to promote cell adhesion and proliferation [5, 6]. Fibrin gel is a degradable biopolymer formed from fibrinogen. Fibrin gel mimics the last step of the blood coagulation cascade and results in a clot of fibrin. Fibrinopeptides are removed from fibrinogen by thrombin [7]. With the changes of conformational structure and the exposure of polymerization sites, fibrin monomers self-assemble into insoluble fibrin gel [8]. The insoluble fibrin gel can be eventually degraded with plasmin-mediated fibrinolysis. The fibrin clot adheres to the native tissue to prevent the leakage of body fluid and provides cell binding sites for cell attachment, migration, and proliferation to promote tissue regeneration [9].

Fibrin gel has been widely used as a bioadhesive in surgeries for hemostasis, wound closure, and a sealant [10–13]. Additionally, fibrin gel exhibits minimal inflammation and foreign body reaction and is readily absorbed during the normal wound healing process. These fibrin sealants have been successfully applied in cardiovascular and neuro- and thoracic surgeries. In recent years, the application of fibrin gel in tissue engineering has become more common. In comparison to the synthetic polymeric materials, fibrin gel presents many advantages, such as controllable degradation rate which matches those of tissue regeneration, nontoxic degradation products, and excellent biocompatibility. Moreover, the morphology, mechanical properties, and stability of fibrin gel hydrogel could be tuned by controlling the precursor concentration and ionic strength [14, 15]. Collagen-based hydrogel, on the other hand, faces the challenge of fast degradation rate, which leads to the instability of mechanical property before the tissue repair or wound healing is done [16]. In addition, fibrin gel also presents high cell seeding efficiency, uniform cell distribution [17], adhesive property [18], and improved cellular interaction [19]. The ability for fibrin gel to cure* in situ* makes it suitable for developing injectable biomaterials that is compatible with minimally invasive delivery approaches. Existing review articles are mainly focused on the use of fibrin gel as a bioadhesive in tissue repair [12, 20–22]. This paper reviews recent advances in applying fibrin gel as an injectable scaffold and cell carrier for tissue engineering.

## 2. Mechanism of Fibrinogen Involved in Blood-Clotting Cascade

**Figure 1:**
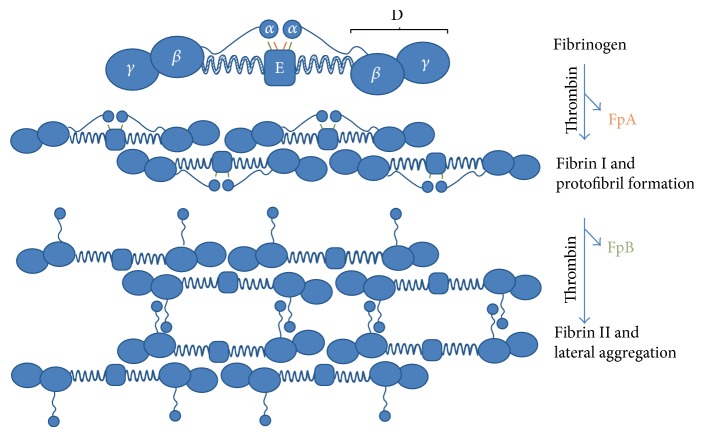
Schematic representation of the fibrin aggregation process. Fibrinogen is composed of two sets of A*α*-, B*β*-, and *γ* chains. Each *α*-chain is connected with E-region through fibrinopeptide A (FPA, orange) and fibrinopeptide B (FPB, green). The D-region is linked with E-region through a coiled segment. Thrombin-mediated cleavage of FPA induces the formation of two-stranded protofibril. Subsequent cleavage of FPB releases *α*-chain from E-region and contributes to the lateral aggregation of two-stranded protofibrils and fibrin formation [24].

Fibrinogen and thrombin are the main components involved in the blood-clotting process. Fibrinogen is a 340 kDa plasma glycoprotein consisting of two sets of polypeptide chains and each set consists of A*α*-, B*β*-, and *γ* chains (Figure 1) [7, 23, 24]. The two sets of polypeptide chains are linked as a dimer by 29 disulfide bonds. B*β*- and *γ* chains consist of the D-region, which is linked with E-region through a coiled segment. *α*-chains are linked to E-region through fibrinopeptide A (FPA) and fibrinopeptide B (FPB). Thrombin is a protease existing in the plasma, which is formed from the proteolytically cleaved prothrombin (coagulation factor II) in the coagulation cascade after the vascular injury [25]. Thrombin-mediated cleavage of FPA and FPB from fibrinogen initiates the formation of fibrin. The removal of FPA occurs first to start the double-stranded protofibril formation. Subsequently, FPB is removed from fibrinogen and results in the release of *α*-chain from the E-region, which leads to a lateral aggregation of protofibrils and fibrin formation. The fibrin continues to self-assemble into a fibrin network.

Fibrin serves as both a cofactor and a substrate for plasmin-mediated fibrinolytic degradation. Fibrin enhances the transformation of plasminogen to plasmin by tissue plasminogen activator (tPA) and breaks down the fiber structure by the cleavage of plasmin in fibrin [24, 26, 27].

## 3. Source and Preparation of Fibrin Gel

Fibrin-based products are prepared from pooled plasma. Human plasma (homologous or autologous) has been used as a source for fibrinogen to reduce the potential risks of immunological reaction [28]. The thrombin is usually purified from bovine plasma. Each of these two precursor solutions is stored in a separate syringe and is mixed and injected directly to the wound site [28]. The gelation process of fibrin gel mimics the last step of the coagulation cascade, which is a part of natural wound healing processes. Fibrinogen is converted to fibrin via the mediation of thrombin. Then fibrin is cross-linked by a coagulation factor and self-assembles into fibrin mesh [29]. Gel formation follows the nonlinear condensation polymerization principle [30]. By changing the kinetic parameters fibrin gel structure can be controlled. For instance, increasing the concentration of thrombin accelerates the gelation time and results in a more densely cross-linked network with thinner fibers. On the other hand, reducing the thrombin concentration results in gel with a higher porosity [30, 31]. Increasing the concentration of FXIIa (a coagulation factor which stabilizes the fibrin) contributes to a denser structure with increased clot stiffness [32]. Fibrin gels with a final fibrinogen concentration higher than 25 mg/mL, 20 mM Ca^2+^, and pH between 6.8 and 9 have a broad linear viscoelastic region. They also present the ability to withstand 10^4^ Pa mechanical load and a long-term stability, which is desirable for tissue engineering application [33]. The degradation rate of fibrin gel can be regulated with aprotinin and tranexamic acid (trans-4-aminomethyl-cyclohexane-1-carboxylic acid; tAMCA) to precisely match tissue regeneration [34]. Fibrin can be fabricated into various types of tissue engineering scaffolds such as micro/nanoparticles [35, 36], micro/nanofibers [37, 38], microtubes [39], and hydrogels [40, 41]. These diverse fibrin-based products have been applied in different tissue engineering fields and some of their recent applications are reviewed below.

## 4. Applications of Fibrin Gel in Tissue Engineering

**Figure 2:**
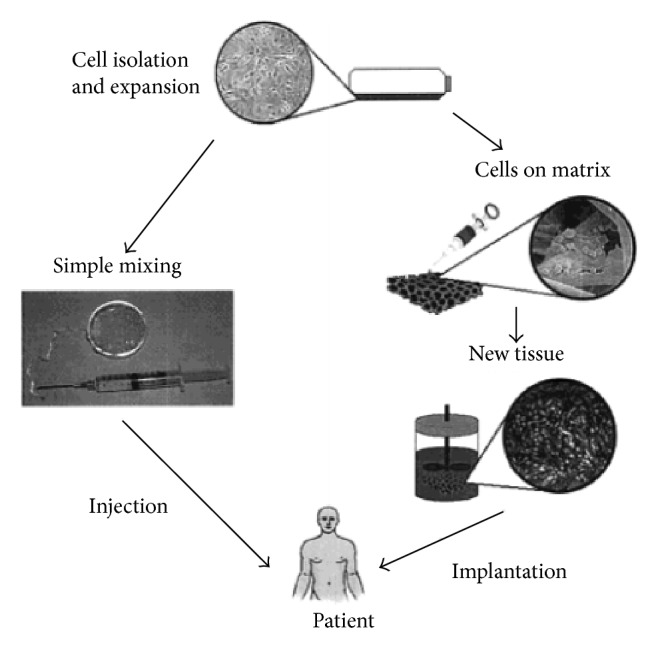
Schematic illustration of two approaches to engineer desired tissue. Cells are isolated from biopsy and mixed with scaffold materials. Subsequently the mixture system is injected into patients' body (left). Alternatively, isolated cells are cultured on a scaffold* in vitro* and implanted into desired place after the formation of new functional tissue (right). Reprinted (adapted) with permission from [42]. Copyright (2001) American Chemical Society.

Tissue engineering is a revolutionary strategy to solve the problem of shortage of donated organ or tissue. Cells are isolated from patient's tissue biopsy and seeded into a scaffold, which provides mechanical support for cell migration, proliferation, and tissue regeneration. There are two approaches to engineer tissues (Figure 2). One of them is to inject the mixture of scaffold precursor and cells into patients' body [42]. The other approach involves culturing the scaffold* in vitro* and implanting the subsequent engineered tissue into patients' body. Occasionally, it is necessary to encapsulate cells in a delivery carrier in order to improve the viability of transplanted cells and tissue regeneration. Therefore, cells will be mixed with delivery carrier first and then the mixture system will be delivered into a scaffold.

### 4.1. Applications of Injectable Fibrin Gel as Scaffolds in Tissue Engineering

**Figure 3:**
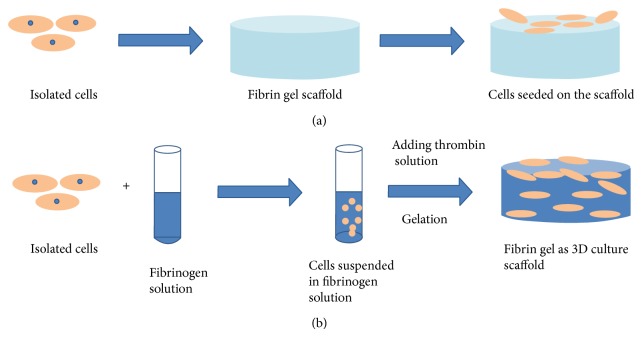
Schematic illustration of fabrications of two- and three-dimensional cell culture scaffold. The conventional two-dimensional scaffold is fabricated in advance of cell seeding and the isolated cells are seeded on the surface of scaffold (a). The three-dimensional scaffold cures in the presence of the encapsulated cells. Then, the mixture can be delivered into a mold to gel or directly injected into a defect in the body (b).

Fibrin gel is able to function as both two-dimensional and three-dimensional cell culture scaffold [43]. As shown in Figure 3(a), the traditional two-dimensional scaffold is fabricated before cell seeding. After the gelation of fibrin gel, isolated cells are seeded into the surface of fibrin gel [44]. Although the conventional two-dimensional scaffold provides an understanding as to how cells interact with the fibrin gel surface, it cannot mimic the natural physiological environment of cells* in vivo*. Three-dimensional scaffolds become popular because of their ability to be a model of tissue physiology and provide a better understanding on the interaction of cell and matrix, as well as how the cell-matrix interaction affects cell function. Moreover, it is essential that such a system has a potential to be developed to engineer functional tissue. Three-dimensional scaffolds are fabricated as described in Figure 3(b). Isolated cells are first suspended in the scaffold precursor solution. Then, the mixture will be delivered into a mold and culture for several minutes to complete the gelation. After the gelation, the construct will be cultured for days for tissue regeneration. Alternatively, the cell-fibrin gel precursor solution mixture can be directly injected into a defect* in vivo* so that the fibrin gel cures and immobilizes cells for the regeneration for the functional tissues. The application of injectable fibrin gel for cardiac and cartilage tissue engineering is introduced.

#### 4.1.1. Application of Injectable Fibrin Gel in Cardiac Tissue Engineering

Coronary heart disease is one of the leading causes of death in the world. The myocardial infarction (MI) causes many irreversible damages to the heart tissue and eventually leads to heart failure [45]. Cardiac transplantation is currently the only option to treat the MI damaged heart tissue. However, due to the shortage of donation researchers have explored tissue engineering method to regenerate functional heart tissues. Christman et al. [45] have demonstrated the feasibility of injecting cell-scaffold mixture into damaged heart after MI to decrease infarct size and improve cell survival. They created MI on female Sprague-Dawley rats through surgery and obtained myoblasts from the hind limb muscle of newborn Sprague-Dawley rats. The isolated myoblasts were suspended in fibrin gel precursor solution and injected into ischemic left ventricle. After five weeks of implantations, the treatment group with cell-fibrin gel mixture attenuated the decrease in thickness of infarct wall and preserve cardiac functions based on histological and echocardiography results, respectively [45]. When compared to direct injection of cardiomyoblasts, fibrin gel was demonstrated to increase the survival rate of transplanted cells, decrease the infarct size, and increase blood flow to the damaged tissue [46].

**Figure 4:**
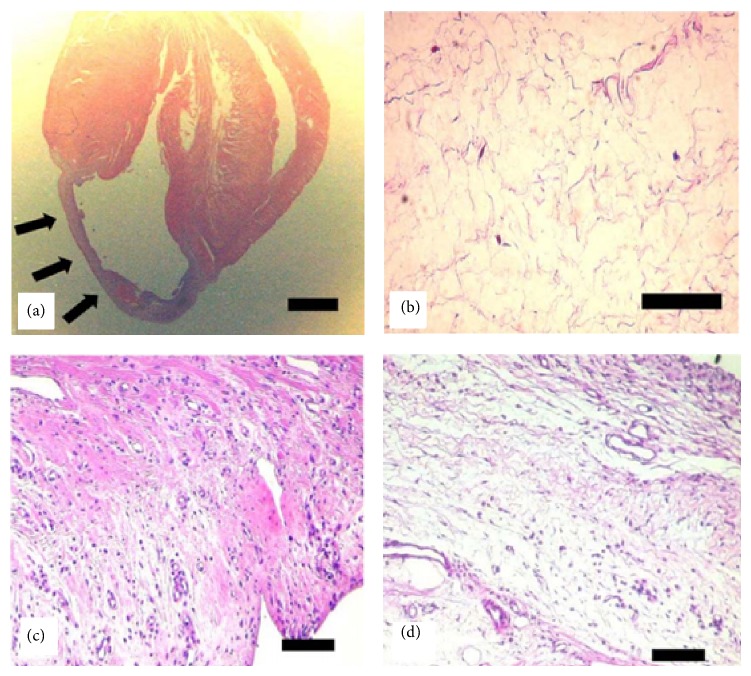
H&E staining of histological tissue section. Myocardium wall became thin in the infarction site (arrows in (a)). No vessels and viable cells were observed in infarction site (b). After eight weeks, the treatment of cell transplantation with fibrin gel (c) demonstrated extensive tissue regeneration when compared with cell transplantation without fibrin gel (d). Scale bar indicates 2 mm (a) and 100 *μ*m ((b), (c), and (d)) [47].

**Figure 5:**
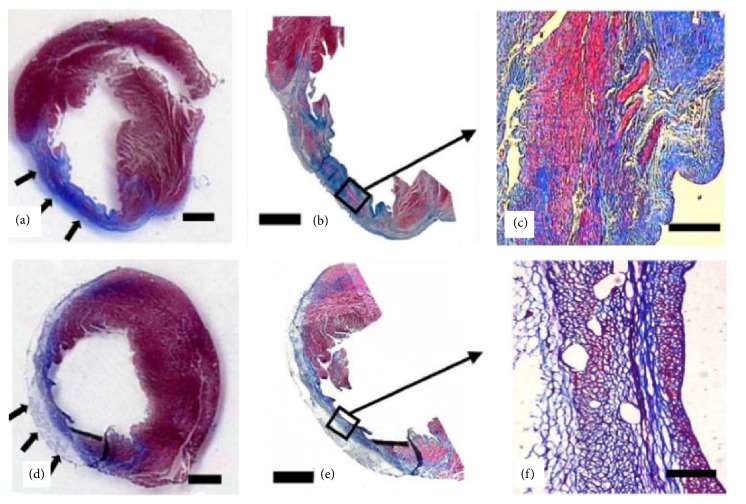
Masson's trichrome staining of infarction site after eight weeks for treatment with bone marrow mononuclear cells delivered with ((a), (b), and (c)) and without ((d), (e), and (f)) fibrin gel. The infarction size of treatment with fibrin gel (arrows) is smaller than the treatment without fibrin gel. Treatment with cell-fibrin gel mixture demonstrated a larger amount of viable tissue (red) and a smaller amount of fibrous tissue (blue) when compared to the direct injection of cells without fibrin gel. Scale bar indicates 2 mm in (a), (b), (d), and (e) and 100 *μ*m in (c) and (f) [47].

Ryu and colleagues [47] injected mixtures of bone marrow mononuclear cells and fibrin gel into the infracted myocardium and found that this formulation enhanced the neovascularization. Results of this study showed that the microvessel density of fibrin gel encapsulated with cells group (350 ± 22 microvessels/mm^2^) was significantly higher than cell-only injection (262 ± 13 microvessels/mm^2^) or medium-only injection (76 ± 9 microvessels/mm^2^). Additionally, the average inner diameter of microvessels of fibrin gel encapsulated with cells group (14.6 ± 1.2 *μ*m) is larger than cell-only injection group (10.2 ± 0.7 *μ*m) and medium-only injection group (7.3 ± 0.5 *μ*m). Hematoxylin and eosin (H&E) staining revealed that the treatment of cell transplantation with fibrin gel resulted in more extensive tissue regeneration in the infarction site when compared to cell transplantation without fibrin gel (Figure 4). Additionally, the infarction site treated with cell-fibrin gel mixture exhibited a larger amount of viable cells and a smaller amount of fibrous tissue compared to the treatment without fibrin gel (Figure 5) [47]. It was also reported that by transplanting adipose-derived stem cells with injectable fibrin scaffolds cell retention was larger than cell-only injection and heart function was also improved significantly [48].

#### 4.1.2. Application of Injectable Fibrin Gel in Cartilage Engineering

Cartilage is a connective tissue with no vascular network in its inner structure. Therefore, it has limited ability to regenerate or repair injured cartilage tissue. Damage of cartilage tissues results in the formation of scar tissues with both structure and function that differ greatly from the undamaged cartilage [49]. Cakmak et al. [50] transplanted chondrocytes with injectable fibrin gel and demonstrated that this approach could achieve cartilage tissue regeneration. They injected chondrocyte-fibrin gel mixture into the forehead and interocular regions of New Zealand white rabbits demonstrated neocartilage formation after eight weeks. Lee et al. [51] reported that by injecting synovium-derived mesenchymal stem cells with injectable collagen/hyaluronic acid/fibrinogen composite gel into rabbit model regenerated and repaired osteochondral defect in knee. Through histological analysis they found that glycosaminoglycans and type II collagen were accumulated within the extracellular matrix. In addition, hyaline-like cartilage construct was produced. After twenty-four weeks, the defects had been repaired with hyaline-like cartilage tissue.

### 4.2. Applications of Fibrin Gel as Cell Carriers in Tissue Engineering

**Figure 6:**
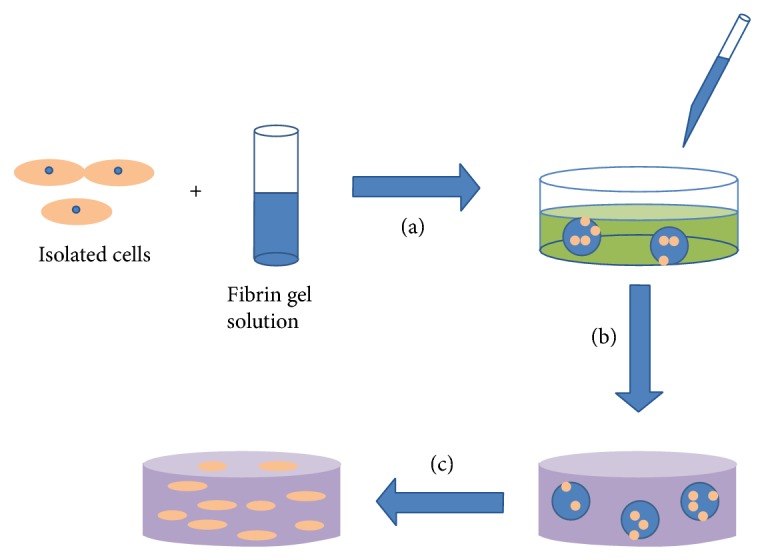
Isolated cells are suspended in fibrin gel solution. The cell suspension is added into cross-linking agent solution dropwise to form microbeads (a). The microbeads are mixed into injectable scaffold solution and injected into a mold (b). The microbeads degrade gradually and leave micropores in the three-dimensional scaffold for the migration and proliferation of released cells (c).

The use of fibrin gel as a cell carrying microbeads has been widely investigated in recent decades. The purpose of using fibrin gel as a carrier to deliver cells into a three-dimensional scaffold is to protect cells from the forces applied during the preparation and delivery processes [52]. Using fibrin microbeads to carry cells results in good cell viability. Isolated cells are suspended into fibrin gel solution (Figure 6). Then the cell suspension will be added to cross-linking agent solution dropwise to form microbeads. Finally, the microbeads will be entrapped into a three-dimensional scaffold for tissue regeneration. The microbeads will degrade gradually and release cells into scaffolds. Additionally, due to the degradation of microbeads micropores will also be left open for cell migration and proliferation [53].

**Figure 7:**
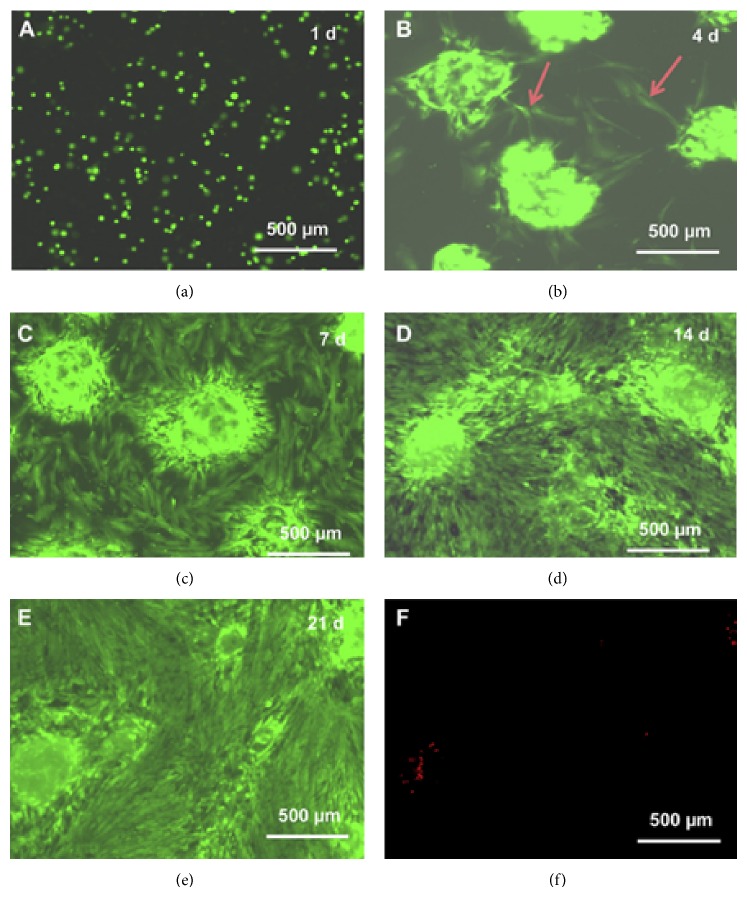
Fluorescent live/dead staining images. Live cells are stained in green and dead cells are in red. Cells were released from the microbeads after 4 days showing healthy polygonal morphology (arrows in (b)). After 7 days, the number of released cells increased greatly. Cells attached to the tissue culture polystyrene and showed a healthy morphology (c). Cells continued to proliferate (d) and formed confluent monolayer at day 21 (e) [53].

**Figure 8:**
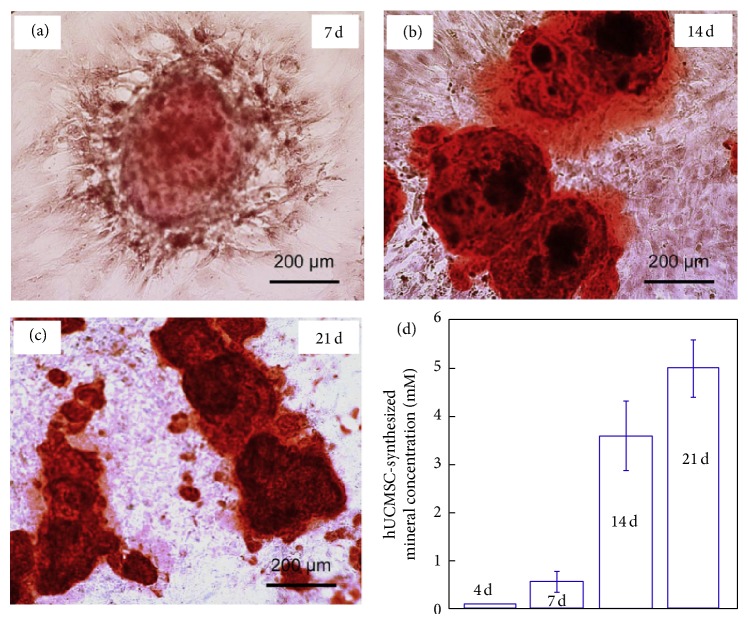
Alizarin staining for the synthesis of bone mineral at 7, 14, and 21 days. The calcium minerals are stained in red. The mineral concentration was measured by osteogenesis assay and the results are shown in (d). The mineral concentration at day 21 is 10-fold higher than day 7, which demonstrated cells released from microbeads synthesized bone mineral successfully [53].

The use of fibrin-based microbeads for stem cell encapsulation and as delivery vehicle along with injectable scaffolds has demonstrated promise in promoting bone regeneration. Zhou and Xu [53] incorporated human umbilical cord mesenchymal stem cells into alginate-fibrin microbeads and added these microbeads to an injectable scaffold. The alginate-fibrin microbeads degraded at day 4 and released the encapsulated stem cells into the scaffold. The released cells showed healthy polygonal morphology and exhibited excellent cell viability (Figure 7). Alizarin staining confirmed the synthesis of bone minerals (Figure 8). Similarly, microbeads-encapsulated stem cells exhibited enhanced cell viability and myogenic differentiation capability for muscle tissue engineering [54]. After 16 days of culture, the percentage of live cells in the microbeads containing scaffold reached 91% and was significantly higher when compared to direct encapsulation of the cells into the construct without the microbeads (cell viability of 81%). The live cell density in the construct with microbeads was also 1.6-fold higher than those without microbeads.

## 5. Future Outlook

Fibrin gel has demonstrated potential in functioning as an injectable scaffold for tissue engineering. However, there are numerous obstacles such as the weak mechanical properties, potential disease transmission, and the shrinkage of the gel that still need to be addressed for the wide adoption of fibrin gel in tissue engineering [55, 56]. It is possible to chemically modify the structure of fibrin gel to improve the mechanical properties and issues associated with gel shrinkage. To improve the mechanical properties of fibrin networks, hybrid composites that combine fibrin with synthetic biodegradable polymers, such as polyglycolic acid [57] and poly(lactic-co-glycolic acid) [58, 59], have demonstrated the ability to promote cell attachment and infiltration as well as tissue restoration. Similarly, fibrin gel formed from genipin cross-linking has demonstrated improved mechanical properties [60]. Genipin-cross-linked fibrin exhibited promise in functioning as an adhesive for repairing intervertebral disc annulus while demonstrating elastic modulus in the range of native annular tissue and remained adhered to the native tissue at strains exceeding physiological levels. Most recently, fibrin gel was functionalized with nitric oxide donors for preparing biomaterials capable of controlling release of nitric oxide for promoting tissue regeneration and wound healing [61, 62].

## 6. Summary

The combination of excellent biocompatibility, controllable degradation rate, adhesive property, and ability to cure* in situ* makes fibrin gel an attractive biomaterial for tissue engineering applications. Fibrin gel self-assembles into a scaffold by mimicking the last step of blood clotting to support cell migration, proliferation, differentiation, and tissue regeneration. It can also be used as cell carriers to protect cells from the forces produced during preparation and delivery processes. Further engineering the fibrin gel through chemical modification can be used to develop tissue engineering scaffolds with improved mechanical properties and multifunctional biomaterials.
